# Comparing the cytotoxicity of taurolidine, mitomycin C, and oxaliplatin on the proliferation of in vitro colon carcinoma cells following pressurized intra-peritoneal aerosol chemotherapy (PIPAC)

**DOI:** 10.1186/s12957-019-1633-5

**Published:** 2019-06-03

**Authors:** Justyna Schubert, Veria Khosrawipour, Haris Chaudhry, Mohamed Arafkas, Wolfram Trudo Knoefel, Alessio Pigazzi, Tanja Khosrawipour

**Affiliations:** 1 Department of Food Hygiene and Consumer Health Protection, Wroclaw University of Environmental and Life Sciences, Wroclaw, Poland; 2 Department of Orthopedic and Trauma Surgery, Ortho-Klinik Dortmund, Dortmund, Germany; 3 Division of Colorectal Surgery, Department of Surgery, University of California Irvine (UCI), California, USA; 4 Department of Plastic Surgery, Ortho-Klinik Dortmund, Dortmund, Germany; 5 Department of Surgery (A), University-Hospital Düsseldorf, Düsseldorf, Germany

**Keywords:** Colon carcinoma, Pressurized intra-peritoneal aerosol chemotherapy (PIPAC), Peritoneal metastasis, Taurolidine, Mitomycin C, Oxaliplatin

## Abstract

**Background:**

Besides its known antibacterial effect commonly used in intraperitoneal lavage, taurolidine has been observed to possess antineoplastic properties. In order to analyse this antineoplastic potential in a palliative therapeutic setting, taurolidine (TN) was compared to mitomycin C (MMC) and oxaliplatin (OX), known antineoplastic agents which are routinely used in intraperitoneal applications, following pressurized intra-peritoneal aerosol chemotherapy (PIPAC).

**Methods:**

An in vitro model was established using a colon adenocarcinoma cell line (HT-29 human cells). Different experimental dosages of TN and combinations of TN, MMC, and OX were applied via PIPAC. To measure cell proliferation, a colorimetric tetrazolium reduction assay was utilized 24 h after PIPAC.

**Results:**

We demonstrated a cytotoxic effect of TN and OX (184 mg/150 mL, *p* < 0.01) on tumor cell growth. An increasing dosage of TN (from 0.5 g/100 mL to 0.75 g/150 mL) correlated with higher cell toxicity when compared to untreated cells (*p* < 0.05 and *p* < 0.01, respectively). PIPAC with OX and both OX and TN (0.5 g/100 mL) showed the same cytotoxic effect (*p* < 0.01). No significant impact was observed for MMC (14 mg/50 mL, *p* > 0.05) or MMC with OX (*p* > 0.05) applied via PIPAC.

**Conclusions:**

The intraperitoneal application of TN is mostly limited to lavage procedures in cases of peritonitis. Our results indicate a substantial antineoplastic in vitro effect on colon carcinoma cells following PIPAC application. While this effect could be used in the palliative treatment of peritoneal metastases, further clinical studies are required to investigate the feasibility of TN application in such cases.

## Background

Intraperitoneal chemotherapy (IPC) has gained increasing acceptance in the past 20 years and has since been frequently used and extensively studied. Limitations concerning drug delivery to solid cancer formations have been a major issue as they contribute to failure in systemic and IPC strategies [1, 2]. It had been argued that, to a large extent, an increased intra-tumoral pressure inhibits the penetration of anti-cancer drugs into these more solid cancer formations [3]. To overcome these limitations, pressurized intra-peritoneal aerosol chemotherapy (PIPAC) has been presented as an alternative option for IPC instead of conventional lavage [4]. Due to good clinical results [5], the current clinical and experimental focus has shifted toward the application of new drugs as well as more complex substances [6–8]. Meanwhile, a drug dosage increase of already applied substances is also under evaluation [9]. While clinical studies are promising, data indicates that there is a relevant amount of patients who do not show any histological regression of their peritoneal metastases (PM). This limited response to PIPAC therapy results in a fast progression of the disease. These patients could benefit from an optimised treatment with taurolidine (TN) administration, which has been indicated as an antineoplastic agent [10, 11]. TN is currently being used in an intraperitoneal application for peritonitis [12–14] by means of lavage. However, limited data is available for its antineoplastic effect in peritoneal cancer, especially when compared to conventional IPCs with, e.g., oxaliplatin (OX) and mitomycin C (MMC), which have been used for peritoneal metastasis (PM) originating from colon carcinoma. The palliative use of TN in PM could be a possibility as was already demonstrated in some animal models [9, 10], especially when exhibiting a similar level of cytotoxicity as in current IPC. If adequate cytotoxicity could be achieved, PIPAC could represent a mean of intraperitoneal taurolidine delivery. During PIPAC, the abdominal cavity is filled with microdroplets in a pressurized environment [15–18]. To investigate whether adequate cytotoxicity of TN is achieved and to evaluate a possible clinical use, we aimed to compare TN at different concentrations to OX and MMC application during PIPAC. Both OX and MMC have been used as a single- or multi-drug treatment of PM. In this study, we used a well-established in vitro colon carcinoma model for PIPAC [19].

## Methods

### Cell cultures

A human colorectal in vitro model was established using a HT-29 cell line. The cell line was obtained from the Institute of Immunology and Experimental Therapy (Wrocław, Poland). HT-29 cells were grown in Dulbecco’s modified Eagle’s medium (DMEM - high glucose, Sigma-Aldrich, Poznan, Poland) supplemented with 10% heat-inactivated fetal bovine serum (FBS, Gibco, Thermo Fisher Scientific, Poland), 2 mmol/L glutamine, 100 IU/mL penicillin, and 100 μg/mL streptomycin (Sigma-Aldrich) at 36^o^C in a humidified 5% CO_2_ incubator. Cells (1.4 × 10^5^ per well) were seeded in 24-well plates (TC Plate 24 Well, Standard, F, Sarstedt AG & Co. KG, Germany) and incubated for 48 h.

### PIPAC model and procedures

The ex vivo PIPAC model has been presented in numerous studies [6, 7]. A temperature of 36 °C was established and continued for the entire procedure by placing the PIPAC box into a heated water bath. Two 24-well plates were positioned at the bottom of the PIPAC box. They were placed lateral of the aerosol jet spray produced by the microinjection pump (MIP®, Reger Medizintechnik, Rottweil, Germany). To further avoid direct exposure of the wells to the aerosol jet, both 24-well plates were placed under a bilaterally open plastic tunnel. The PIPAC box was then hermetically closed. A CO_2_ capnoperitoneum was created within the box and continued for the entire application. TN (Taurolin® Ringer 0.5%, Berlin-Chemie AG, Berlin, Germany), MMC (Sigma-Aldrich), or OX (Medoxa, medac GmbH, Wedel, Germany) was applied onto the exposed tumor cells in aerosolised form.

### Drugs doses

In current literature, the dosage of OX used for PIPAC has been described as 92 mg/m^2^ body surface. This is delivered via 150 mL of 5% glucose solution. The solution is aerosolized in a capnoperitoneum of 12 mmHg. This dosage has demonstrated a significant cytotoxic effect in PIPAC application [19]. The calculations of drug volume and concentration of MMC were based on the data available for OX. We used 14 mg of MMC in 50 mL of 0.9% saline solution with 10 % addition of DMSO (Sigma-Aldrich), which provided a full drug solubility. TN was applied in 3 different doses: 0.25 g, 0.5 g, and 0.75 g dissolved in 50, 100, and 150 mL, respectively. To evaluate the effect of a single-drug versus multiple-drug treatment on tumor cell toxicity, the following options were tested: for a single-drug PIPAC, MMC/OX/TN-0.25 g/TN-0.5 g/TN-0.75 g, and for a multi-drug PIPAC, OX + MMC/OX + TN-0.5 g.

### Exposure time

After 48 h of incubating the HT-29 cells, the culture medium was removed and replaced with 150 μL of fresh medium. Thereafter, PIPAC was performed in 2 steps. First, TN or MMC was applied followed by OX. Cells were exposed for an additional time of 30 min after PIPAC. Drug-treated cells were incubated at 36^o^C with 5% CO_2_. Following the period of exposure, all medium, including drug solution, was aspirated from the cells and replaced with fresh medium. Cells were incubated for 24 h at 36^o^C and 5% CO_2_. Then, the MTS proliferation assay was performed.

### MTS test

A colorimetric CellTiter 96® AQ_ueous_ One Solution assay (Promega, Poland) was used to measure cell proliferation 24 h after PIPAC. The test was performed according to the manufacturer’s instruction with modifications. Briefly, the medium was removed from each well and replaced by 0.3 mL of fresh DMEM. Next, after 1 h of incubation at 36^o^C at 5%CO_2_, an MTS-based reagent was added to each well and absorbance at 490 nm was detected using a microplate reader (Tecan, Basel, Switzerland). The untreated cells were used as a control group. For all groups, the percentage of proliferation was correlated to the control group.

### Statistical analysis

Experiments were performed three times. All wells were counted without exclusion. To compare the independent groups, the Kruskal-Wallis analysis of variance on ranks was performed. Probability (*p*) values were defined as *p < 0.05, **p < 0.01, and ^#^*p* > 0.05, with a *p* value <0.05 to be statistically significant. Data is shown as the mean standard deviation.

## Results

### Effect of single-drug PIPAC on colon tumor cells growth

**Fig. 1:**
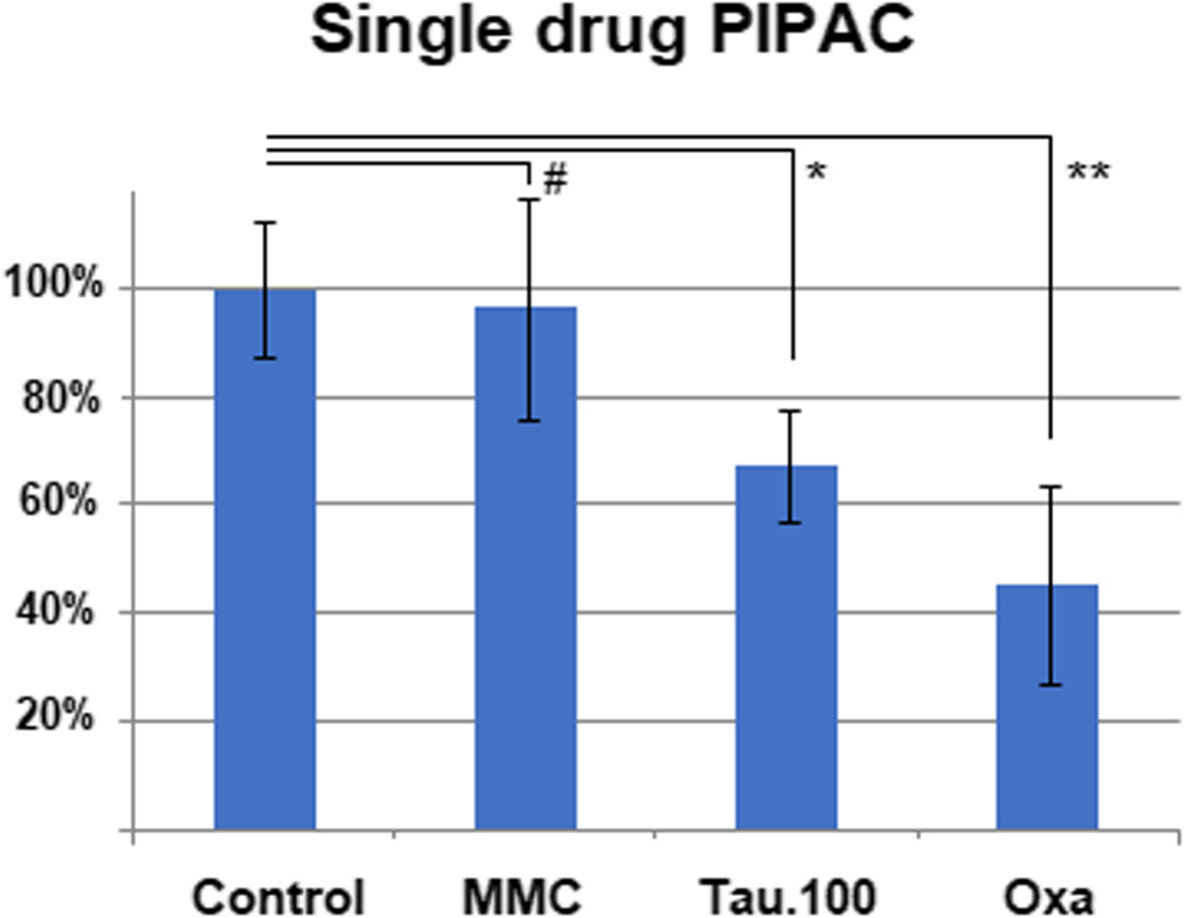
Effect of single-drug PIPAC (mitomycin C 14 mg/50 mL, taurolidine 0.5 g/100 mL, and oxaliplatin 184 mg/150 mL) on colon carcinoma cell toxicity

**Fig. 2:**
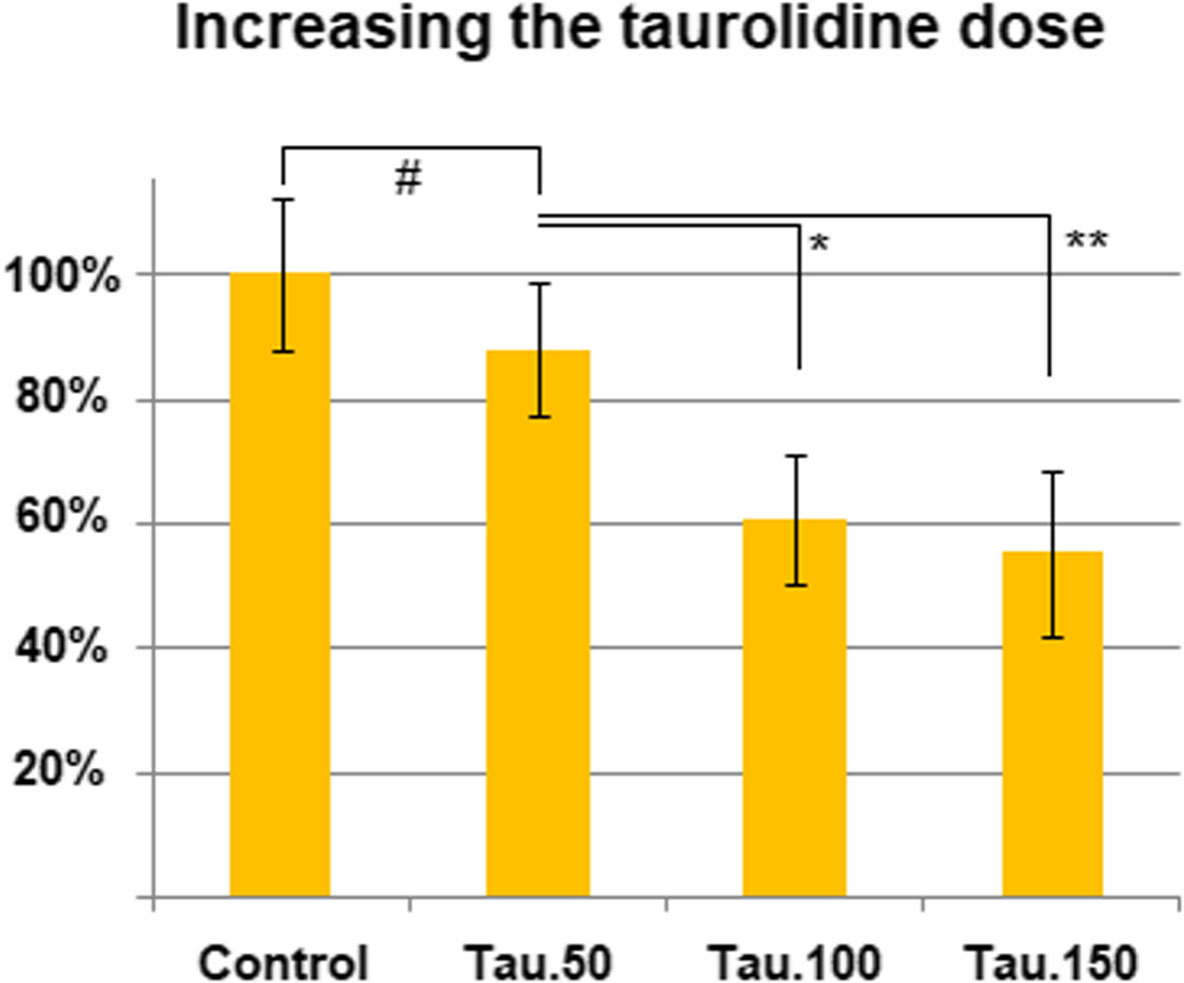
Effect of taurolidine dose escalation (0.25 g/50 mL, 0.5 g/100 mL, and 0.75 g/150 mL) on colon carcinoma cell growth

PIPAC procedures were performed without major difficulties. Moreover, it was technically possible to apply TN despite its detergent properties as a liquid and its foam-creating characteristics. Among tested drugs that were incubated with HT-29 cells, TN (0.5 g/100 mL), and OX showed the most potent inhibition of cell growth when compared to untreated cells (*p* < 0.05 and *p* < 0.01, respectively). No significant effect was observed for MMC (*p* > 0.05) (Fig. 1). However, the inhibitory effect of TN was dose dependent. The lowest applied dosage of TN (0.25 g/50 mL) did not exert any significant impact compared with the untreated control group. However, an increase in dosage correlated with higher tumor cell death. The cytotoxicity grew from 0.5 g to 0.75 g TN compared to the untreated control group (*p* < 0.05 and *p* < 0.01, respectively). The results of the TN dose escalation are summarized in Fig. 2.

### Effect of multi-drug PIPAC on colon carcinoma cell growth

**Fig. 3:**
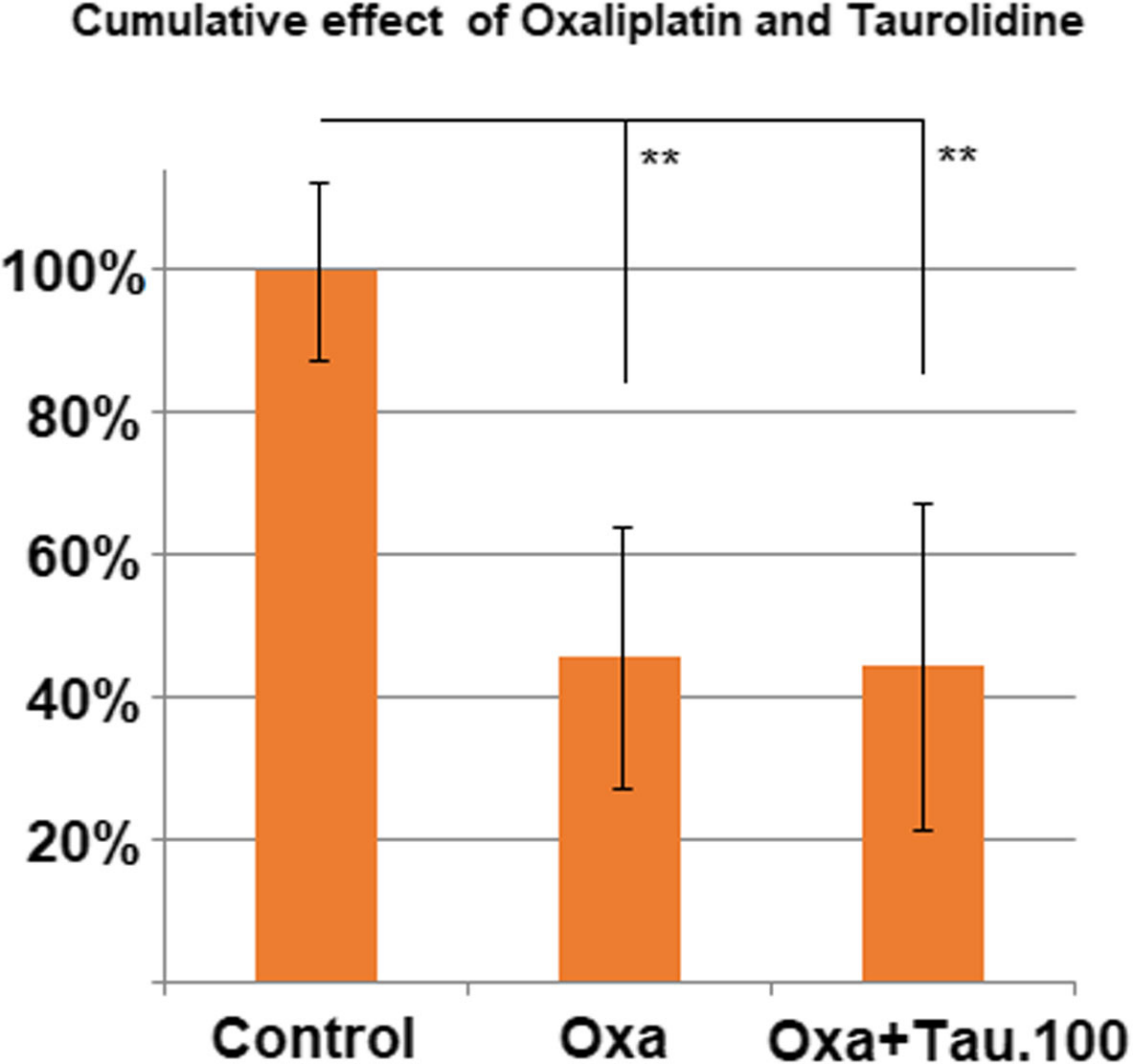
Effect of combination treatment with oxaliplatin (184 mg/150 mL) and taurolidine (0.5 g/100 mL)

**Fig. 4:**
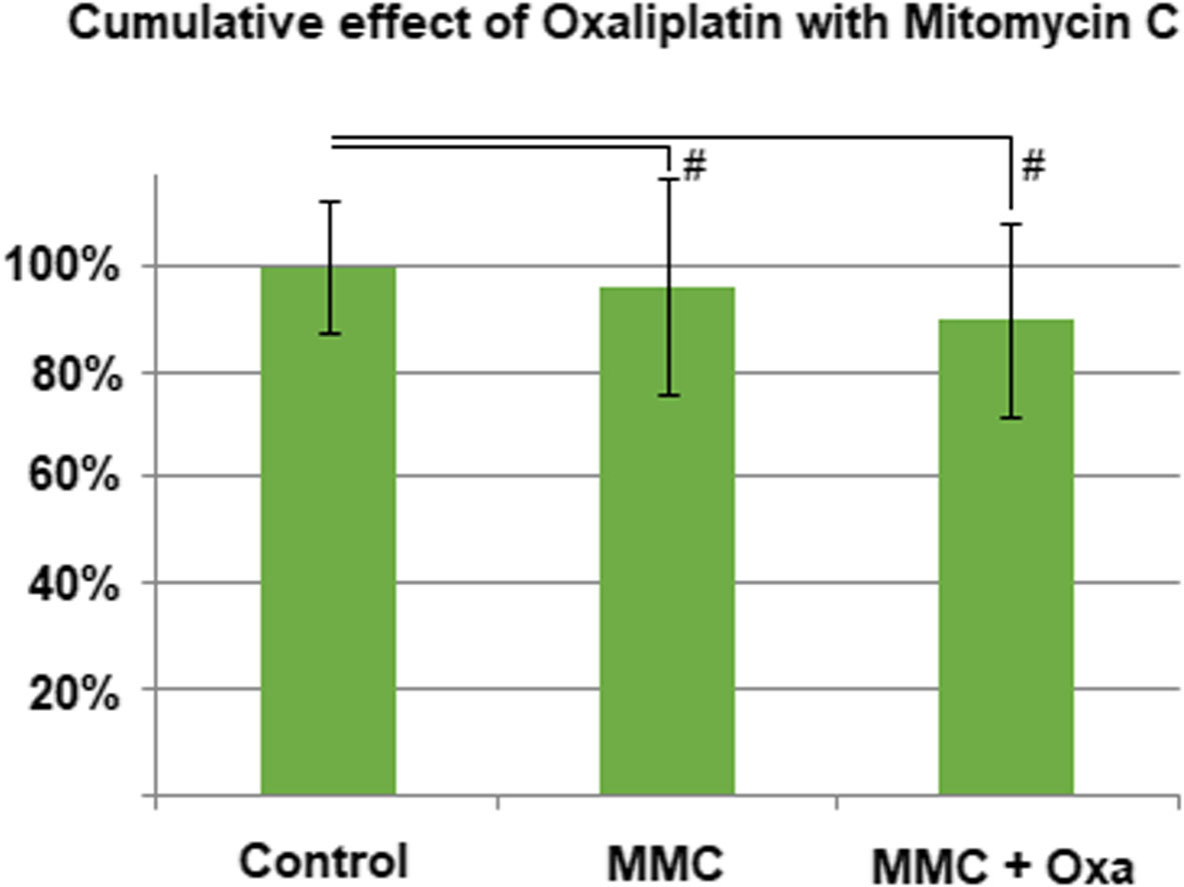
Effect of combination treatment with oxaliplatin (184 mg/150 mL) and mitomycin C (14 mg/50 mL)

The combination of OX and TN did not show any increase of cytotoxicity versus OX alone. Compared to the untreated control group, there was no significant difference between PIPAC conducted with only OX and treatment augmentation with TN (0.5 g/100 mL). In both cases, the proliferation of cells was inhibited by approximately 50% (*p* < 0.01, Fig. 3). Similar results were observed in the multi-drug combination of OX and MMC. The combined application of MMC and OX did not show significantly higher cell toxicity (*p* > 0.05) when compared to the untreated control group (Fig. 4).

**Fig. 5:**
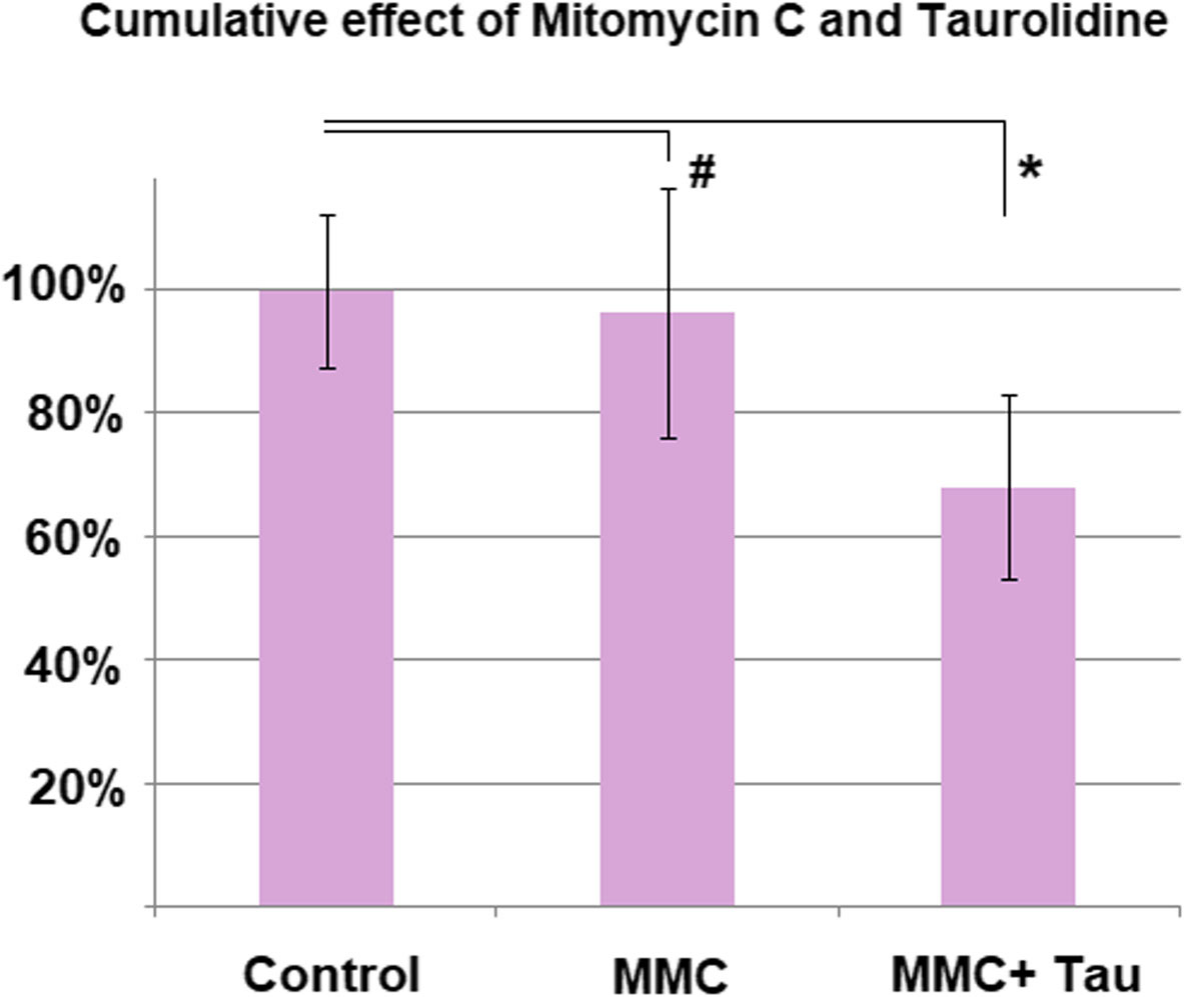
Effect of combination treatment with taurolidine (0.5 g/100 mL) and mitomycin C (14 mg/50 mL)

Although the combined application of MMC and taurolidine resulted in significantly higher cell toxicity (*p* < 0.05) when compared to the MMC alone (Fig. 5), the combination of both drugs had similar results as taurolidine alone.

## Discussion

The search for new drugs and drug combinations for intraperitoneal applications has been ongoing [20–22]. The introduction of PIPAC has increased the interest in new substances which could improve overall cytotoxicity. The intraperitoneal cavity allows for the application of some substances that cannot be applied intravenously due to their toxicity or limited efficacy [10]. Nevertheless, while many possibly new substances are available, there has been little clinical experience on these drugs. In contrast, TN is a substance that is clinically used in intra-abdominal surgery due to its antibacterial effects. There are some basic studies on its antineoplastic properties after its first use as an antiseptic agent, especially by Jacobi et al. [23]. So far, the clinical use of TN in PM has been neglected due to the availability of other, more established chemotherapeutic substances, such as OX and MMC. Since TN’s overall potential as an antineoplastic agent has been scarcely studied comparing its effects to known agents is presents challenges. Our experimental data confirm the antineoplastic activity of TN previously described by other authors [10, 11, 14] and compares this effect with current agents applied via PIPAC. Our findings further confirm previous recommendations which favour OX over MMC in the treatment of colon carcinoma [24–26]. Although MMC shows cytotoxicity on colon carcinoma cells, this effect seems to be far less than expected especially in comparison to OX and taurolidine. This effect has also been documented in clinical studies [26]. Data also indicate that the combined use of OX and MMC might possibly interfere with their overall efficacy and reduce their respective cytotoxic effects due to possible interactions. OX is known to exhibit pharmacological instability [27] as well as significant interference with other drugs [28], which might explain some of the observed effects. However, these data must be interpreted with caution as an in vitro cell experiment displays some limitations with respect to in vivo pharmacokinetics and possible influence on the immune system. A significant improvement for IPC could be reached using TN monotherapy or in combination with OX as an auxiliary treatment. Based on these data, more clinical studies are required to evaluate TN application’s safety and efficacy as well as possible toxicity in the treatment of PM. However, at least theoretically a clinical benefit from using OX as an auxiliary drug can be assumed.

## Conclusion

TN shows a significant cytotoxic effect when applied with PIPAC and should be evaluated in further clinical studies. The cytotoxic effect of the low doses applied here is similarly effective to that of standard doses of oxaliplatin currently used. This might especially be of high value in cases of chemoresistant PM after multiple cycles of PIPAC.

## Data Availability

Our data is freely available if any scientist wishes to use them.

## Abbreviations

CG: Control group
CO_2_: Carbon dioxide
DMSO: Dimethyl sulfoxide
IPC: Intraperitoneal chemotherapy
MMC: Mitomycin C
MTS: 3-(4,5-dimethylthiazol-2-yl)-5-(3-carboxymethoxyphenyl)-2-(4-sulfophenyl)-2H-tetrazolium
OX: Oxaliplatin
PIPAC: Pressurized intra-peritoneal aerosol chemotherapy
PM: Peritoneal metastasis
TN: Taurolidine
